# We-Care-Well: exploring the personal recovery of mental health caregivers through Participatory Action Research

**DOI:** 10.3389/fpubh.2024.1366144

**Published:** 2024-04-04

**Authors:** Tyler Redublo, Sayani Paul, Anahita Joshi, Simone Arbour, Ross Murray, Mary Chiu

**Affiliations:** ^1^Research & Academics, Ontario Shores Centre for Mental Health Sciences, Whitby, ON, Canada; ^2^Translational Research Program, Temerty Faculty of Medicine, University of Toronto, Toronto, ON, Canada

**Keywords:** personal recovery, family and friend caregivers, mental health, Participatory Action Research, CHIME framework, recovery college model

## Abstract

Family caregivers play a critical role in supporting the recovery journeys of their loved ones, yet the recovery journeys of family caregivers have not been well-explored. Using a Participatory Action Research approach, we explore the personal recovery journeys of family caregivers for individuals with mental illness. This case study involved piloting and exploring the impact of a novel online workshop series offered to mental health caregivers at Ontario Shores Center for Mental Health Sciences. Recovery courses and workshops conventionally engage patients living with mental health conditions. In the current case, the recovery model is adapted to the needs and experiences of their family caregivers, resulting in a pilot workshop series called “We Care Well”. Through participant-led discussions, interactive and take-home activities, and experiential learning, caregivers co-created workshop content and engaged in peer-learning on seven personal recovery-oriented topics. This included: self-care, resilience-building, non-violent communication, storytelling, and mental health advocacy. Throughout the sessions, participants implemented their learnings into their caregiving roles, and shared their experiences with the group to progress through their own recovery journeys. The We Care Well series was found to be an effective intervention to adapt and apply the personal recovery framework to mental health caregivers. PAR, and co-design are viable approaches to engage caregivers in mental health research, and can facilitate knowledge exchange, as well as relationship building with peers and program facilitators.

## 1 Introduction

In this Community Case Study, we explored the recovery journeys of mental health family caregivers - denoted here as “caregivers” - through a series of workshops titled, “We-Care-Well” (WCW). The workshops were delivered at Ontario Shores Center for Mental Health Sciences (thereafter referred to as “Ontario Shores”), a specialized mental health hospital offering a range of mental health services and recovery-oriented care to those living with mental illnesses and their family members and caregivers. The focus of the workshops was on personal recovery in mental health. Personal recovery is a deeply personal process whereby individuals embrace a sense of purpose and connection to live a meaningful life, despite the limitations caused by the condition (1, 2).

Caregivers who provide care for individuals with mental health challenges experience their own stressors and challenges, which puts them on their own unique recovery journeys. To date, there has been a lack of personal recovery-oriented research and initiatives that are focused on the needs and experiences of caregivers. The current study aims to fill this gap in knowledge and practice by applying the personal recovery model to mental health caregivers. As such, the objectives of this project were to pilot and implement a recovery-based program for caregivers. Specifically, we adopted Participatory Action Research (PAR) and Narrative Analysis approaches, to develop a case study which provides supporting evidence for why and how the personal recovery model is not only relevant for individuals with mental illness, but instead, can also be applied to the caregiver population. This research is couched within the overall purpose to support caregivers' recovery journeys.

Personal recovery-oriented research for caregivers is essential because it places the caregiver at the center of their healing process, fostering empowerment, hope, and holistic wellbeing. Research allowing caregivers to acknowledge the uniqueness of their experiences has the potential to transform their journey and promote a positive and inclusive approach to mental health.

## 2 Context and background

### 2.1 The role and experiences of caregivers

In 2021, the International Alliance of Carer Organizations estimated that there were 7.8 million caregivers in Canada, and 56.4 million caregivers in the United States (3). The effort and time provided by caregivers relieves the costs and burden on social and healthcare systems, by providing health services in our communities and homes (3). However, the time demands of caregiving significantly affect caregivers' quality of life and their wellbeing, and can limit their opportunities for leisure, social interaction, exercise, and self-care (4, 5). Further, adult caregivers have higher rates of heart disease and depression, among other physical and mental health conditions (6–15), compared to non-caregivers.

Caregivers play an instrumental role toward the support and recovery of their loved ones and care recipients. However, caregivers themselves are rarely acknowledged for the unique mental health experiences, challenges and stressors that they face in their role. They may encounter challenging emotions in their role, including hopelessness, anger, fear, shame and loneliness (16–22). Feelings of inadequacy and a sense of loss for themselves and their loved ones (19) are also commonly shared amongst caregivers. As such, caregiving has a significant impact on one's psychosocial and mental health, and there is a need to build support for caregivers' own personal recovery.

### 2.2 Applying personal recovery to a novel population

Personal recovery has been defined as the process of developing attitudes, values, feelings, goals, skills, and/or roles that contribute to a satisfying, hopeful, and generative life, despite limitations caused by illness (22). Personal recovery is distinct from clinical recovery, which focuses more on symptom management, and a return to functional baseline (23, 24). Both of these unique processes significantly contribute to an individual's state of health, wellness and quality of life.

Personal recovery is the primary focus of Recovery Colleges, which were introduced over a decade ago in the United Kingdom to complement traditional and more clinically based mental health services (25). Recovery Colleges consist of a roster of courses that individuals with mental health challenges choose to attend to learn about a certain topic, and also to get peer support. Courses are typically informed by service users' interests, including applied life skills (e.g., *Living on a Budget*). Traditionally, recovery courses are co-produced and delivered by individuals with lived experience (e.g., with mental illness) and a professional on the topic (e.g., social worker, dietitian or artist). These courses build resiliency and community engagement (25).

In the mental health domain, the CHIME (Connection, Hope, Identity, Meaning, and Empowerment) framework (26) typically underpins the development of recovery practices and recovery college models (1, 27, 28). CHIME is a comprehensive theoretical framework for understanding personal recovery in adults with mental health conditions. In this project, the CHIME framework was used as a guiding tool in the development of workshop-related activities for caregivers.

Caregivers play a critical role in supporting the recovery journey of their loved ones, yet there is limited research examining how to support caregivers' own wellbeing, strength and resilience (17, 19, 21). To date, the majority of work on caregivers' personal recovery has been centered around sharing their “stories” amongst peers (21). While this approach provides many benefits (e.g., better coping, building resilience, reducing negative feelings by focusing on the positive aspects of their roles), simply sharing experiences does not offer holistic support nor does it effectively address caregivers' needs (20). Further, story sharing can place an overemphasis on the burdens and challenges of caregiving which can overshadow the strengths and proficiencies they have developed (19). This highlights the need for peer-driven and strength-based programming for caregivers. For instance, peer support in mental health and trauma-informed care is an effective means of shifting from a biomedical model to recovery-oriented principles. It affords the development of relationships rooted in mutual respect, shared experiences, and empowerment and fosters hope (29). As such, programs that focus specifically on personal recovery for caregivers are long overdue.

## 3 We-Care-Well series

### 3.1 Objectives

We-Care-Well (WCW) is a novel initiative developed and facilitated at Ontario Shores from January to October 2023, three series of biweekly, virtual workshops were facilitated via videoconferencing platform *Zoom* (30). Each workshop series offered caregivers in local communities the opportunity to recognize, learn more about, and support their own wellbeing, by progressing through their recovery journeys. The three **key objectives** of the series were to provide caregivers with:

1. Knowledge of recovery-oriented principles to promote and reinforce self-care, resilience, and strengths in caregivers.
2. Approaches to leverage recovery perspectives and principles to navigate around, or even navigate “through” challenges and barriers within the caregiving role.
3. Practical, actionable skills and strategies that support their own mental health and wellbeing.

### 3.2 Methodological approaches: Participatory Action Research and narrative analysis

**Figure 1:**
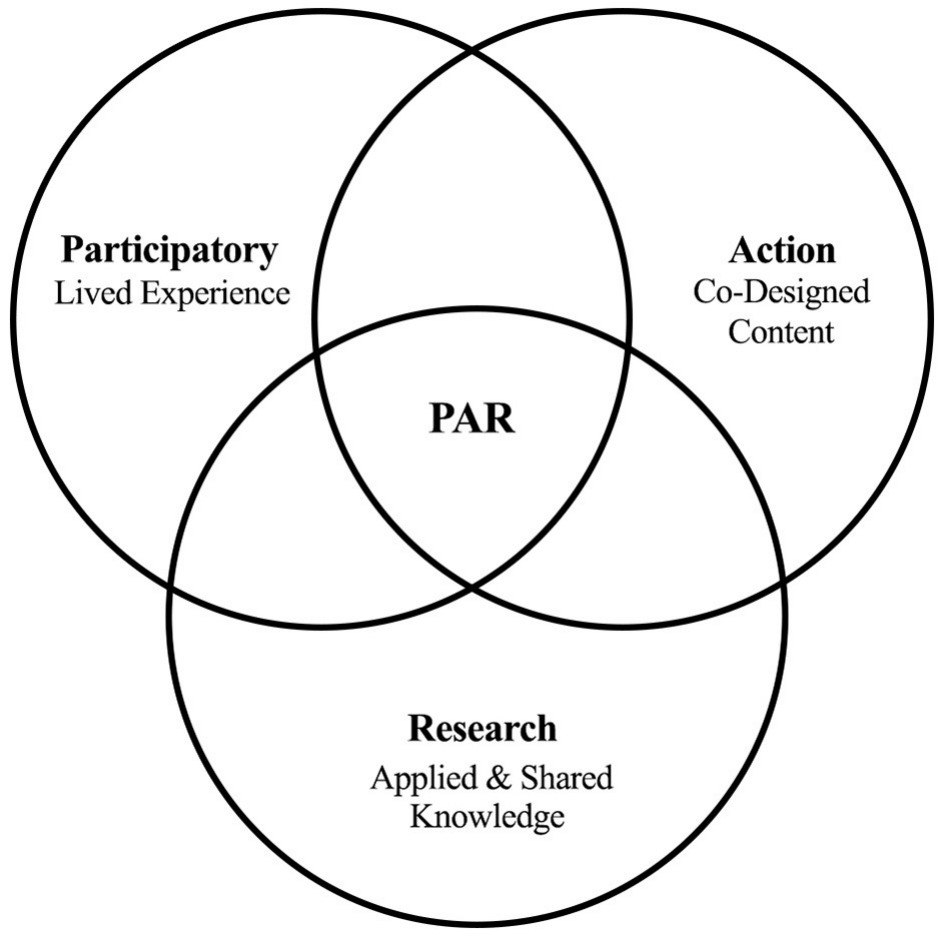
Conceptual diagram illustrating the three pillars of Participatory Action Research as it pertains to We-Care-Well, and the products of their overlap. Retrieved and adapted from Chevalier and Buckles (32).

Development of WCW's workshop content was guided by principles of Participatory Action Research (PAR), a methodology that involves an iterative process of inquiry, reflection, and action to improve health. It focuses on reducing health inequities and decentralizing traditional research by involving those most affected (31). PAR (Figure 1) is grounded within three pillars (33):

- Participation, which is the meaningful, genuine and democratic engagement with individuals with lived experience;
- Action, which involves the application of tangible, direct practices that improve the human experience and/or personal wellbeing;
- Research, which refers to the advancement and integration of information and knowledge. All three pillars must be working synchronously to effectively facilitate PAR.

Additionally, a narrative analysis approach (34) was adopted to guide an ongoing, creative process of sensemaking. Narrative analysis is uniquely beneficial toward personal recovery as it emphasizes connecting experiences and actions from the past to the present, thus creating meaning that is unique to each person's story. It also brings clarity to caregivers' recovery as an interconnected, purposeful experience of seemingly distinct and unrelated events. Lastly, it provides opportunities for transformation and healing (35), despite the challenges associated with being a mental health caregiver.

### 3.3 Workshop content and participants

WCW workshop topics were chosen through a series of needs assessment and brainstorming sessions with family caregivers and representatives from *Ontario Shores Recovery College, Family Council* and *Family Resource Center*. Selected workshop topics aligned with the CHIME framework—Connection, Hope, Identity, Meaning, Empowerment—and pertain to the intersection of personal recovery and caregiving (see Table 1). Participants were first introduced to the “Caregiver Recovery” as a novel concept and encouraged to explore, as a caregiver community, how the Recovery model and CHIME Framework—traditionally applied to individuals living with mental health and addictions—may be integrated into their own caregiving journeys.

**Table 1:** Objectives and descriptions of We-Care-Well: recovery workshop series for caregivers.

**Overall workshop series objectives**		
- Learn about recovery-oriented principles to promote and reinforce self-care, resilience, and strengths as family caregivers.		
- Understand how to leverage recovery perspectives and principles to navigate around, or even navigate through challenges and barriers within the caregiving role.		
- Share practical, actionable skills and strategies that support mental health as caregivers.		
**Topic**	**Description**	**Key discussion question(s)**
Caregiver recovery	Participants are introduced to the CHIME Framework—Connection, Hope, Identity, Meaning and Empowerment—for personal recovery (26) and explore with each other the different ways to integrate these elements in their caregiving journeys.	• *What does “recovery” mean to you?* • *What are some challenges and barriers you face as a caregiver?* • *What self-care strategies do you use?* • *What type of communication strategies do you use in your role as a caregiver?*
Non-violent communication (NVC) in caregiving	Participants learn about the Non-violent Communication model (36) and how to apply it in their caregiving role. Guided by a trained-practitioner, participants practice the four principles of NVC: Observation, Feeling, Need, and Request.	• *4-step process of the nonviolent communication model:* ○ *Observation* ○ *Feeling* ○ *Need* ○ *Request*
Mental Health Act and your role as the substitute decision maker	Facilitated by a Bioethicist, participants discuss and explore how to advocate for themselves and the persons they are caring for, in the mental health care system by demystifying the Mental Health Act.	• *As caregivers, how do we navigate making difficult decisions for our care recipients?* • *What are your personal core values? How do these apply as powers of attorney or substitute decision-makers?*
Compassionate care	The focus of this workshop is on self-care and self-compassion as a holistic practice. Literature has shown that self-compassionate people tend to elicit increased care and support in their relationships with others (37). Here participants share strategies on how to care for physical, emotional, mental, spiritual, financial, and social wellbeing.	• *As caregivers, how can we acknowledge our feelings, and practice self-compassion & self-care?* • *Has anyone experienced compassion fatigue? What was that experience like?*
Creative storytelling	Participants are introduced to different approaches to express and share their recovery journeys and lived experiences with other family members and caregivers e.g., Photovoice, which is a health promotion and research approach that empowers caregivers to “identify, represent, and share their realities/experiences through their photos”. This offers fellow caregivers and other individuals the unique opportunity to “see” the relevant challenges from the caregivers' viewpoints (38), and elicit conversations that may bring about changes.	• *If you were writing a book about yourself, what would the title be?* • *Why is storytelling important for Personal Recovery?* • *How can we effectively create and tell our recovery stories?*

A semi-structured guide was developed by TR and MC for each workshop, which served as a guide for facilitation. The workshop content was developed iteratively, based on participant interactions, verbatim dialogue, and experiences shared. This allowed participants to inform and co-create workshops.

WCW workshop series were listed on the *Ontario Shores Recovery College Curriculum; https://www.ontarioshores.ca/resources-support/recovery-college, which is open to the public*. Individuals were invited to register for an account through the *Ontario Shores Recovery College Registration Portal*, which is hosted by MedSIS. Description of the WCW workshop series for each term may be viewed and downloaded through this portal. All community members who identify as providing care for someone living with a mental health condition may self-register for the series. Inclusivity, accessibility and anonymity are key guiding principles of the Recovery College. Thus, every WCW workshop series operates on a self-screening and self-selection process, and no demographic information is collected from the participants. Workshop activities involved interactive discussions on various topics relating to participants' caregiving experiences, perspectives, and needs. Discussions were facilitated by various team members using the online collaborative whiteboard tool *Miro*. Other aspects of workshop programming included icebreaker activities, group reflections, as well as providing practical resources for individuals (e.g., take-home activities).

To ensure and maintain the fidelity of the intervention, team members facilitated debrief meetings immediately following workshops, in which key findings from the session were discussed, feedback was consolidated, as well as the identification and delegation of next steps. Moreover, planning meetings were held 1–3 days before each workshop to conduct a walkthrough of proposed programming, coordinate team member roles, and perform a final check of required resources and materials.

### 3.4 Research ethics exemption

Research ethics exemption was obtained for this study from the Ontario Shores Joint Research Ethics Board (JREB # 22-036-P). Proposed activities were considered Program Evaluation and Quality Improvement and were exempted from JREB review in accordance with the Tri-Council Policy Statement or the Ontario Shores JREB requirements.

## 4 Findings

A total of 17 caregivers self-registered and participated in WCW, across three separate workshop series, spanning from January to October 2023. All participants were women, lived in the community, and provided care to someone living with a mental health condition. Findings of this case study represent a combination of participants' contributions and responses to session activities, team observations, and empirical outcomes across three workshop series. Field notes and post-group debrief meeting notes were reviewed to give rise to four themes: Environment of Trust and Rapport, Participant Led Discussions, Caregiver Identity and Application of Knowledge.

### 4.1 Environment of trust and rapport

The team took deliberate steps to create an environment of trust, and rapport, allowing participants to feel comfortable being vulnerable, and to share their personal stories safely. At the outset of each workshop, facilitators outlined a Comfort and Membership Agreement (see Appendix A) before any recovery-oriented discussions commenced. These functioned as ground rules that established important principles such as respecting diversity of experiences and opinions, and maintaining confidentiality of workshop activities.

Finally, the facilitators provided various mental health supports and resources, in the case of participant crises or emergencies. One important disclaimer that the team stated was that the WCW workshop series was *not* treatment and should not be substituted for clinical care. In the case of a mental health crisis or severe distress, the team provided the phone number for a central intake and crisis hotline, as well as contact information for other family resources.

### 4.2 Participant led discussions

All three workshops were primarily centered on participant-led discussions, in which the facilitators posed open-ended questions for all participants to provide their ideas and responses to. A breakdown and summary of the main discussion questions from each workshop can be seen in Table 1. These questions became vehicles for caregivers to reflect on and share their lived experiences.

To maximize participation and the richness of information shared, free-flowing, dynamic discussions were encouraged. Facilitators ensured that conversations were participant-led, and engaged caregivers as partners. One exemplar scenario of participant-led discussions during this series was when one participant expressed difficulties in finding enough time to focus on self care. She acknowledged that time management is an important skill to develop, however she stated, “*I don't know what I should take out of my current routine to make time though*.” In response to this, various caregivers offered their own insights and experiences. One participant mentioned intentionally incorporating self-care activities into routines, such as engaging in deep reflection, while taking a shower. Another participant recommended setting important boundaries to prioritize time for oneself, which involves learning how to become comfortable saying “no”.

In this way, the directions of the discussions were influenced by the participants themselves, while facilitators managed and guided the processes of knowledge exchange to align with the personal recovery model. By incorporating their own unique needs, values and insights into the discussions, the participants molded the workshop activities in ways that made sense to them, fit their worldviews, and aligned with their recovery.

### 4.3 Serial and iterative design of workshops

All pilot workshop series consisted of three to five workshops; each of which were iteratively designed and built upon the learnings from the previous session. Given this serial design of WCW, the team carefully curated and framed all workshop activities and discussions for participants to conceptualize their recovery as a journey and process. For example, in one workshop, caregivers discussed various forms of communication strategies that they applied within their caregiving role, including- but not limited to- communicating with family members and friends, healthcare practitioners, and directly to their care recipient. Using the *Miro* board, the team captured the verbatim responses provided by participants, including: “*Acknowledging you don't have all answers- build trust*”, and “*Don't impose or force ‘coming from a humble place*”'. A total of 12 different responses were collected, which can be seen in Appendix B.

Following this workshop, the team used narrative analysis (34) to consolidate caregiver responses into four key themes regarding their communication strategies: *(1) Compassion and Understanding Others, (2) Establishing and Maintaining Trust, (3) Humility*, and *(4) Seeking Out Resources and Practical Support* (see Appendix B). From there, presenting the four themes back to the caregivers served to validate their strategies of communication, by making sense of their experiences, and relating them back to their recovery journeys.

Moreover, through this process, participants were primed to conduct deeper, more meaningful explorations of these same topics in subsequent sessions. For example, participants explored how to communicate their needs to different audiences such as healthcare practitioners and other family members, or more nuanced skills like non-violent communication as outlined by Lee at al. (36). As demonstrated here, the serial and iterative structure of the workshops afforded both continuity and flexibility of activities, and further complimented the co-design process as caregivers actively shaped their own learnings.

Another example of an iterative process through a reflection-action cycle was observed during the workshops focused on gratitude, where participants self-identified their skills and strengths in the caregiver role. Caregivers were guided in strength-based reflections, and stated traits they were thankful for such as being a good listener, creative, understanding. To transition from reflection to action, the participants were then presented with strategies on how to express gratitude on a daily basis. This included a take-home activity inspired by (39) Seligman et al.'s various gratitude-oriented interventions and writing a letter to someone in their life who they were grateful for. Finally, to reset the cycle of action and reflection, participants shared their experiences implementing the activities at subsequent sessions.

### 4.4 Caregiver identity

A significant, recurring theme observed throughout WCW was that mental health caregivers face unique, identity-related challenges to their personal recovery, such as feelings of guilt. Many caregivers cited feeling responsible for the wellbeing and health status of the persons they care for, to the point that it became a significant obstacle toward their own wellbeing. Some participants described their guilt as something that they had to “*get over*” and “*overcome…so that I can feel good about things that are supposed to make me happy*”. One participant even reflected on the negative effects of caregiver guilt as being “*self-sabotaging*”.

The significant influence of the caregiver role on these individuals' identities was also observed in the introductory workshops of each series. Most individuals naturally gravitated toward sharing their caregiver personas as part of their introductory statements. Even though unprompted, many openly shared the context and story of their caregiving experiences, such as the mental health diagnoses of their loved ones, as well as their specific caregiving responsibilities.

Most attendees also had a tendency to prioritize the needs of their care recipients before themselves. Many tied their understanding of “recovery” to seeing their loved ones in a happy and prosperous state, even at the expense of their own self-care. One participant went so far as to say that “*…if my son is not doing well, I'm not doing well…*” Another participant admitted that it did not come naturally to ask herself. “*What about me?*” thus leading to consequences of her “*learning the hard way*” to be more attentive to her own needs.

### 4.5 Application of knowledge

In WCW, caregivers are encouraged to apply workshop learnings into their daily caregiver encounters. Workshops served as the key components of co-learning, participation and group reflection. Then, following each session, individuals were provided with practical exercises to apply what they learned to their own unique caregiving contexts. As such, the person-centered impact of PAR extended beyond merely a conceptual understanding of personal recovery, but instead enabled tangible opportunities to bring theory into practice. For example, during a workshop on self-care strategies, participants were guided to collectively brainstorm self-care ideas (e.g., baking, walking exercises- for full list see Appendix C). In a subsequent workshop of the series, the focus shifted toward ideating actionable steps to apply these strategies into practice. Caregivers proposed incorporating self-care into their daily routines through “*Habit stacking”*, “*Setting boundaries so that [they]can make time for [themselves]”* and providing themselves with incentives and rewards for carrying out self-care activities.

Following each workshop, facilitators provided participants with take-home activities to incorporate personal recovery concepts into their caregiving roles. In the aforementioned workshop focused on self-care, attendees were provided with a Wellness Toolbox, sourced from the Canadian Mental Health Association (40). The toolbox was a digital resource that functioned as a guide to organize a wellness plan. It included a list of holistic comfort strategies, stepwise processes to conduct reflection practices, as well as a daily plan to track routines and habits related to wellness and recovery goals. This can be found in Appendix D.

## 5 Discussion

Overall, PAR as a methodological framework, enabled the process of personal recovery to be meaningfully integrated into the WCW series. Facilitators adhered to principles of PAR throughout workshop delivery, which included power sharing, leveraging strengths and opportunities, and honoring the lived experience and diverse perspectives from caregivers. The following section outlines how the three pillars of PAR were conceptually aligned with the recovery journeys of caregivers, and practically applied in the WCW series.

### 5.1 Participation (P)

To create an environment of active participation in WCW, the team applied several strategies. The first was to ensure that workshop facilitators (EM, CA, and TR) had lived experience in caregiving. Consistent with the principle of recovery-oriented care that highlights the importance of peer-led initiatives, WCW facilitators with lived experience of caregiving acted as “peers”. This contributed to rapport and trust building, dispelling power dynamics, and an environment of open dialogue. As a result, facilitators were able to further expand upon discussions and knowledge shared by drawing on their own unique experiences. Doing so helped establish trust and a sense of community amongst the workshop attendees.

Another factor that contributed to workshop participation was the delivery format. Conducting the sessions virtually afforded greater convenience and flexibility, which was especially useful for caregivers who were working through busy schedules, and multiple competing priorities. Further, the team used digital platforms that enabled multimodal forms of synchronous participation and communication, namely *Zoom* and *Miro*. Using these platforms, ideas and responses could be shared in various ways: verbally, textually, and directly through the *Miro* whiteboard. Similarly, Guay et al. (41) found that both multimedia and interactive online activities were two important components of effective internet-based interventions for caregivers. Incorporating these into WCW allowed the team to collect rich responses in the participants' own words, maintain engagement through live interaction and real-time updates to the *Miro* board. This approach also facilitated inclusivity for individuals who preferred to provide their ideas textually.

Finally, participation was enabled by offering various opportunities for caregiver feedback and self-reflection. For instance, embedded throughout each workshop were reflection periods, where caregivers shared their novel and salient learnings from the session. These presented opportunities to further solidify what they learned, and to identify opportunities to tangibly apply them to their daily routines. Participants also used this time to share any constructive feedback on workshop programming, such as determining which topics and sub-topics may be relevant to them. Similar to what was reported in Dupuis et al. (42), sustaining meaningful participation for caregivers throughout each workshop series was crucial for the purposes of actively reflecting on, questioning, and interpreting the experiences shared. Doing so also avoided a wholly prescriptive design of programming, and provided a sense of continuity of lessons learned. Finally, it played a pivotal role in identifying optimal ways to craft and disseminate their personal recovery journeys, while taking actionable steps based on their acquired insights.

### 5.2 Action (A)

Using PAR, caregivers “actioned” on their own mental health stressors, through (1) acknowledging the challenges they face within their caregiving role, and (2) collectively with their fellow caregivers, identifying and experimenting with solutions that work for them, and repeating this process. Consistent with PAR, participants in WCW took their learnings beyond the workshops, into action (33). Following each session, individuals were provided with practical exercises to apply what they learned to their caregiving roles. As such, the person-centered impact of PAR extended beyond merely a conceptual understanding of personal recovery, but instead enabled tangible opportunities to bring theory into practice.

One application of the reflection-action cycle was on the topic of gratitude. Gratitude is defined as the emotional experience of identifying, focusing on, and appreciating the positive aspects of one's life (43). In one study on Australian mental health caregivers, it was found that those who focused on what they have, as well as those who appreciated others, experienced lower levels of caregiver burden. Moreover, research has demonstrated that expressing gratitude can improve general wellbeing (43, 44). As such, gratitude plays a vastly relevant role toward personal recovery. During workshops focused on gratitude, participants self-identified their skills and strengths in the caregiver role, and shared their experiences how they practiced expressing gratitude during subsequent sessions.

“Identity” is one of five principles of the “CHIME Framework of Personal Recovery” (26). The main objective is to guide participants in overcoming stigma and regaining a positive sense of self and identity (26). Individuals living with mental health conditions and addictions have attempted to articulate the “loss of self” they experience as “stealing me from myself” (45). For caregivers, they may experience role captivity, whereby they feel entrapped by their caregiver role and lose their self-identity outside of caregiving. In other words, they may sacrifice their other identities and roles as caregiving becomes the predominant role. Role captivity (46) may be related to feelings of guilt, inevitability, isolation, and loss of control in caregivers (47, 48). During WCW workshops, participants had the opportunity to explore and reflect on their self-identities and learn strategies that would empower them in their caregiver role, as well as practical actions that would allow them to reevaluate and reclaim their own identities (e.g., self-care, self-compassion, delegation, socialization).

In terms of caregivers' application of workshop lessons, one notable observation was the high degree of self-awareness and deep reflection demonstrated by the caregivers. On most occasions, they were able to articulate their needs and reflect on their experiences and challenges without assistance. However, they may lack a broader framework to conceptualize and understand their experiences in a holistic way. As a result, caregivers had a natural proclivity toward integrating and adapting how the elements of CHIME framework applied to their lives. Overall, caregivers were able to connect concepts of personal recovery to their participation in WCW. Consequently, they were able to integrate and utilize the workshop learnings through caregiving actions in their daily lives, then map those onto aspects of the CHIME framework.

### 5.3 Research (R)

The R in PAR refers to knowledge creation. Through facilitation of the workshop series, facilitators were able to co-create and iteratively integrate knowledge based on caregivers' narratives and sharing of their lived experience into subsequent workshop content development. Reflection time was intentionally embedded in each workshop so that caregivers may have ample opportunities to share their views and understanding of personal and mental health recovery, and how these may positively impact them in the caregiving role.

Following each workshop series, the team reviewed participants' responses to workshop activities, such as screenshots of *Miro* board online discussions and recorded field notes of participant dialogue and interactions. Using this narrative analysis approach led to tangible changes to WCW overtime, such as expanding workshop topics to include caregiver advocacy, and the Mental Health Act. Moreover, ongoing evaluation and quality improvement efforts allow for the development of a dynamic, contextual and continuously evolving concept of personal recovery, as it pertains to caregivers.

Piloting this novel initiative served dual functions, both as program evaluation and quality improvement for future caregiver-oriented offerings at Ontario Shores Recovery College. The knowledge gathered and lessons learned from each WCW series have been used to inform and improve the subsequent iteration of the series, including the upcoming program in January 2024. This enables the continual exploration and operationalization of personal recovery in caregivers and broad scaling of this model to other mental health service delivery organizations.

## 6 Recommendations

Underpinning the WCW workshops using the PAR framework highlights three important recommendations for the development of future workshops. First, PAR encourages a cyclical process of reflection and action. Throughout the workshop series, the team encouraged caregivers to self-reflect (e.g., on feelings, learnings, and experiences) and subsequently share their ideas externally with the group. This reflection led to the brainstorming of practical strategies and actions, that were implemented by individuals between workshops, and repeated in a cyclical process. As such, this process is a critical component when developing and adapting programs using a personal recovery model.

Second, in contrast to the conventional researcher-research subject power dynamic, using PAR, both parties collaborated to create knowledge together. To achieve this with WCW, facilitators had current and/or past experience as caregivers and presented themselves as such to the attendees. This helped establish trust, where individuals could feel comfortable sharing their caregiving experiences among peers. Further workshops would likely benefit from adapting this type of collaborative approach in the development and dissemination of knowledge.

Third, PAR encourages caregivers to adopt active roles where they can shape the workshop series to align with their own recovery journeys. With support from our team, the flow of information and creation of knowledge was primarily led by the caregivers, whereby subsequent workshops were iteratively developed based on the discussions, and ideas shared by attendees. Overall, these reasons highlight how the PAR framework was foundational to the development of the WCW series. We recommend including PAR, and specifically these ingredients for success, as a framework for the development of future workshops.

## 7 Considerations and future directions

While this case study represents a significant step toward exploring personal recovery in mental health caregivers, there are noteworthy limitations. A total of 17 caregivers participated across three workshop series, with the range of five to seven participants at each series. Typically, to enable meaningful exchange and dialogue between participants in group interventions, a range of 2–14 participants are recommended, with an ideal number of 8–12 individuals (49). For future iterations of WCW, expanding the number of participants can provide insight as to whether this model can be broadly scaled, as well as inform a standard rate of attendance for optimal engagement and participation.

There was also a lack of diverse representation of participants (e.g., all participants were female). Caregivers represent an extremely heterogeneous population with unique caregiving responsibilities, demographic variables, and cultural norms. The processes of co-design and knowledge exchange may vary based on differences from minority gender, ethnocultural, and sociodemographic populations. As such, it would be prudent to explore if and how various demographic and identity-related factors influence the acceptability and uptake of this personal recovery model in unique caregiver populations. Applying an equity, diversity, and inclusivity lens throughout participant recruitment, engagement, and workshop content development, are all strong starting points to further explore the diverse experiences of mental health caregivers.

Finally, to transition this individual case study to more robust, comprehensive, and wide-reaching caregiving-oriented programming, it will be important to conduct empirical research and evaluate the long-term impact of WCW interventions. For instance, collecting pre- and post-intervention data on key metrics, such as those aligned with the CHIME framework of personal recovery. In this regard, it is critical to first define and contextualize improvements in personal recovery for caregivers. A scoping review was conducted on various self-reporting measures to assess the personal recovery of caregivers of people living with psychosis (50). They found that no single measure or instrument sufficiently and comprehensively assessed personal recovery in caregivers. This highlights the need for better data collection tools that can accurately measure personal recovery in caregivers, considering factors such as guilt, resilience, confidence, and identity in the caregiving role. With improved methods of data collection, important data-driven decisions can be made for personal recovery-oriented interventions for caregivers, and will further substantiate the need for tailored resources, co-designed by and for this important population.

## 8 Conclusion

The current paper presented the WCW workshop as a novel approach to empower mental health caregivers in acknowledging their important contribution within their role as caregiver, and emphasize the importance of exploring their own recovery journeys. Caregivers are encouraged to apply knowledge of recovery concepts, and actively practice self-discovery and identity-formation in their role as caregivers. The personal recovery-oriented model provides caregivers with the opportunity to be active contributors in the co-design process. This research contributes to the understanding of caregivers' diverse experiences which is essential for creating a more comprehensive and effective healthcare and support infrastructure, which caregivers urgently need. The findings of this workshop series may enable the future development of evidence-based, inclusive, and personalized supports and resources that meet the unique needs of the diverse caregiver population. This case study presents a blueprint for other community and healthcare organizations to create and adapt personal recovery-oriented programming specifically for caregiver populations, with the goal of building capacity and strengthening a broader community of caregivers.

## Data availability statement

The raw data supporting the conclusions of this article will be made available by the authors, without undue reservation.

## Author contributions

TR: Conceptualization, Formal analysis, Project administration, Supervision, Writing – original draft, Writing – review & editing. SP: Conceptualization, Writing – original draft, Writing – review & editing. AJ: Writing – original draft, Writing – review & editing. SA: Supervision, Writing – review & editing. RM: Writing – review & editing. MC: Conceptualization, Project administration, Resources, Supervision, Writing – original draft, Writing – review & editing.
