# Supplementary material for: We-Care-Well: exploring the personal recovery of mental health caregivers through Participatory Action Research
Source: Front Public Health. 2024 Apr 4;12:1366144. doi: 10.3389/fpubh.2024.1366144 (PMC11024292; doi:10.3389/fpubh.2024.1366144)

**Supplementary material**

**Appendix A. Comfort and Membership Agreement.** List of workshop rules and expectations provided to WCW attendees at the outset of each session.

1. *“Vegas Rule”. Who you see here, what you hear here, when you leave here, please let it stay here. Respect the privacy of other members and hold anything said in class as confidential*
2. *Share the space*
3. *If you need to take a break or are feeling uncomfortable, please feel free to, and you can reach out to us for support, as well*
4. *Please inform us if you wish to withdraw or are going to miss a workshop*
5. *Please mute yourself during the presentation and use the ‘Raise Hand’ icon if you wish to speak*
6. *Do not attend classes if you have taken alcohol or drugs*
7. *Respect diversity and differences of opinions and experiences*
8. *Treat everyone the way you would like to be treated*
9. *Participate in creating an environment that is safe, supportive and helpful for learning*
10. *Please refrain from voicing any personal or controversial opinions regarding religion or politics*
11. *We encourage everyone to share, but please note this is not a therapy group, so emergencies must be brought to your clinician*
12. *Ask for clarification if there is anything you are unsure about*

**Appendix B. Caregivers’ Communication Strategies and 4 Resulting Themes.** Left column: caregiver responses to the discussion question, *“What communication strategies do you use in the caregiving role?”* A total of 12 responses were collected. Right column: 4 themes of communication synthesized from caregiver responses, using narrative analysis. Responses are linked to their respective communication theme(s) using black lines.


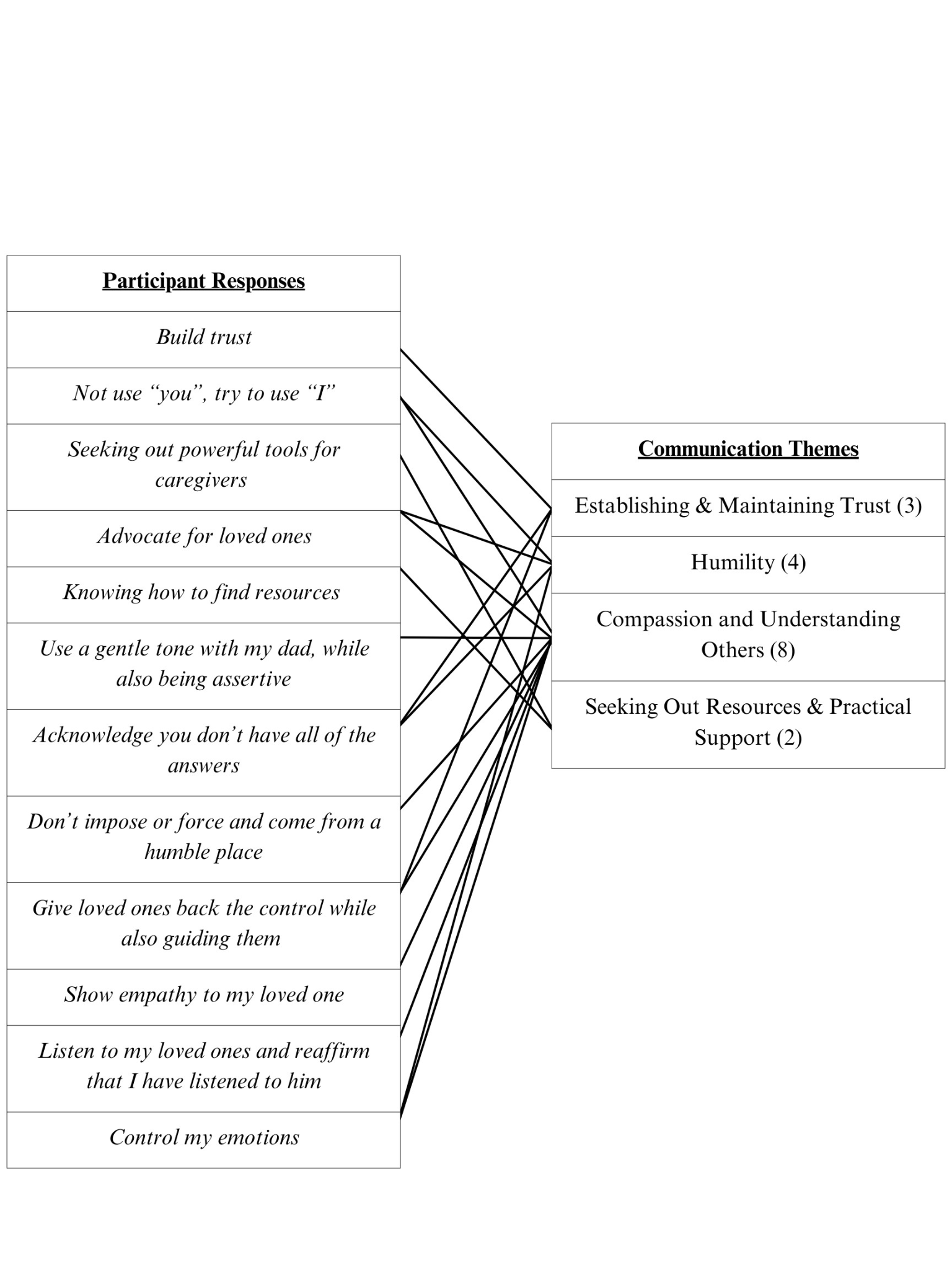


**Alternative Format for Appendix B:**

| **Participant Responses** | **Communication Themes** |
| --- | --- |
| *Build trust; Acknowledge you don’t have all of the answers; Giving loved ones back the control, while also guiding them* | Establishing & Maintaining Trust |
| *Not use ‘you’, try to use ‘I’; Advocate for loved ones, Acknowledge you don’t have all of the answers, Control my emotions* | Humility |
| *Not use ‘you’, try to use ‘I’; Advocate for loved ones, Use a gentle tone with my dad, while also being assertive, Don’t impose or force, and come from a humble place, Give loved ones back the control while also guiding them, Show empathy to my loved one, Listen to my loved ones and reaffirm that I have listened to them, Control my emotions* | Compassion and Understanding Others |
| *Seeking out powerful tools for caregivers, Knowing how to find resources* | Seeking Out Resources and Practical Support |

**Appendix C. Participant Responses to Question: “*What self-care strategies do you use in your role as a caregiver?”*** A total of 19 responses were collected. The image was captured as a screenshot of the online *Miro* board used for Workshop #1 in WCW series #1.


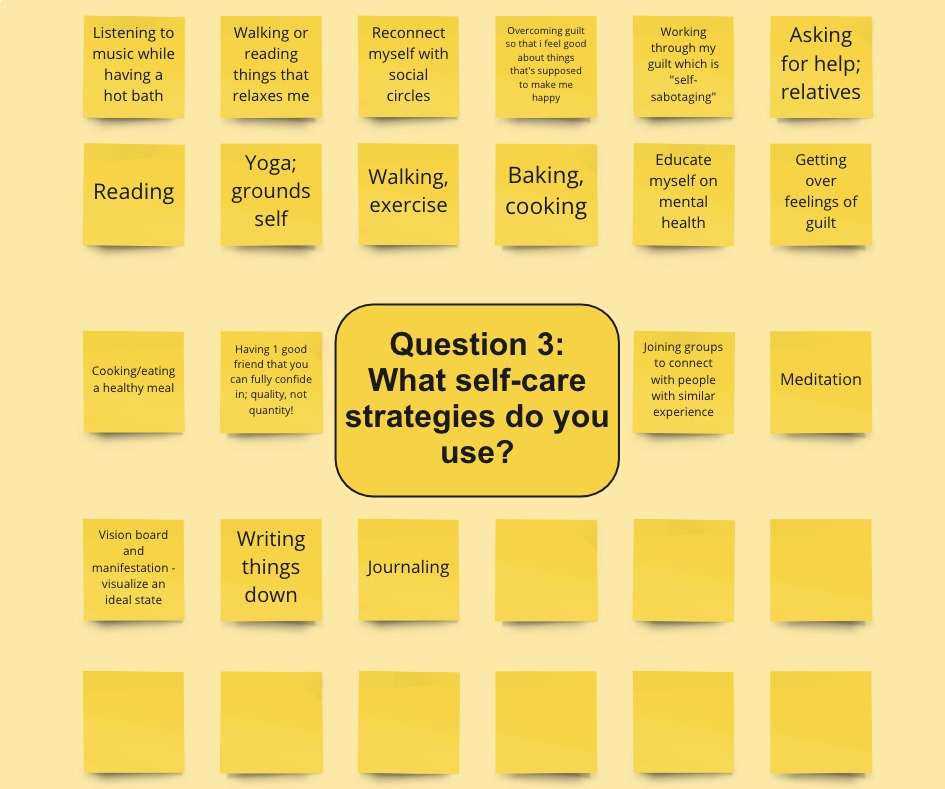


**Appendix D. Wellness Toolbox resource.** A guide to organize a personal wellness plan for caregivers. Sourced from the Canadian Mental Health Association [[37](https://paperpile.com/c/SprZUW/dKeh)].


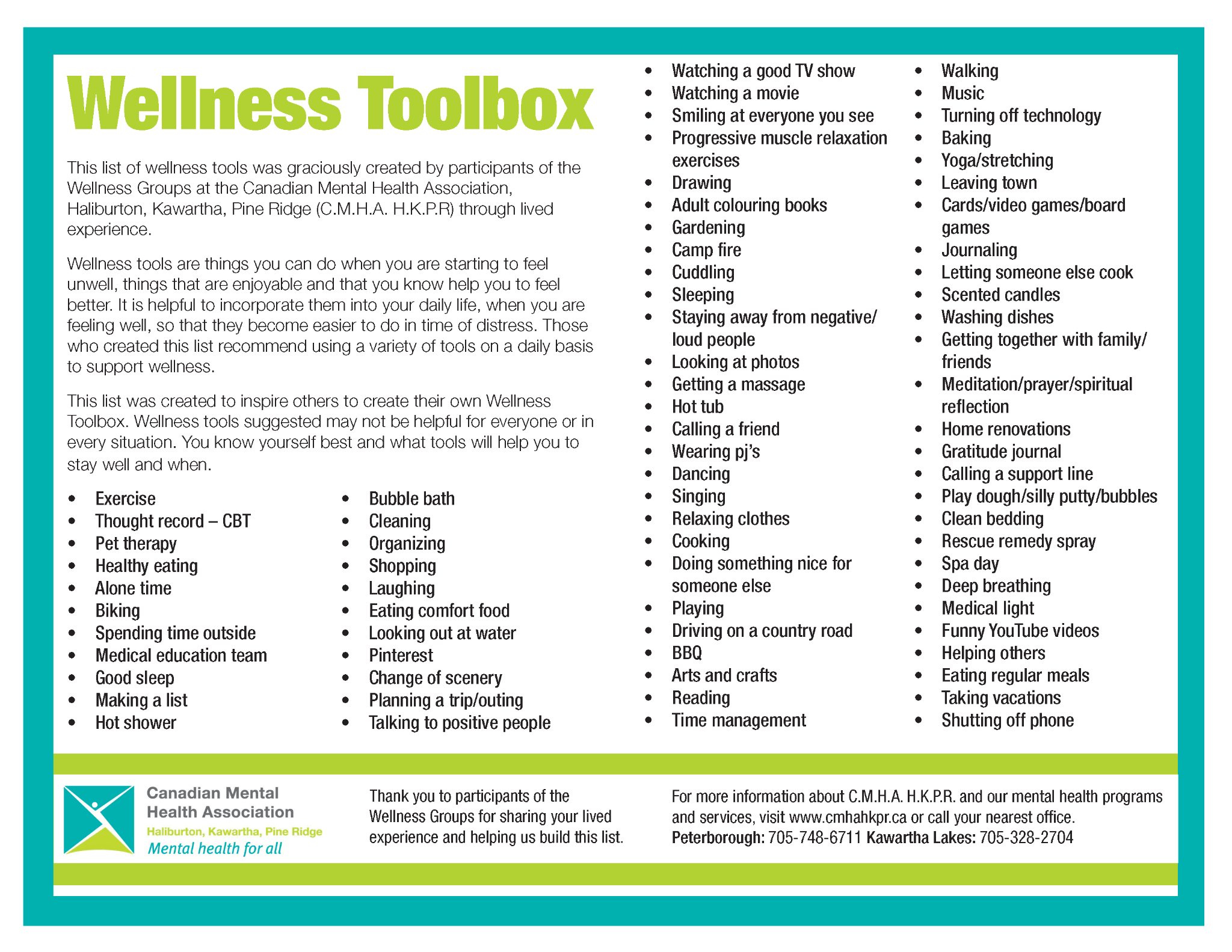

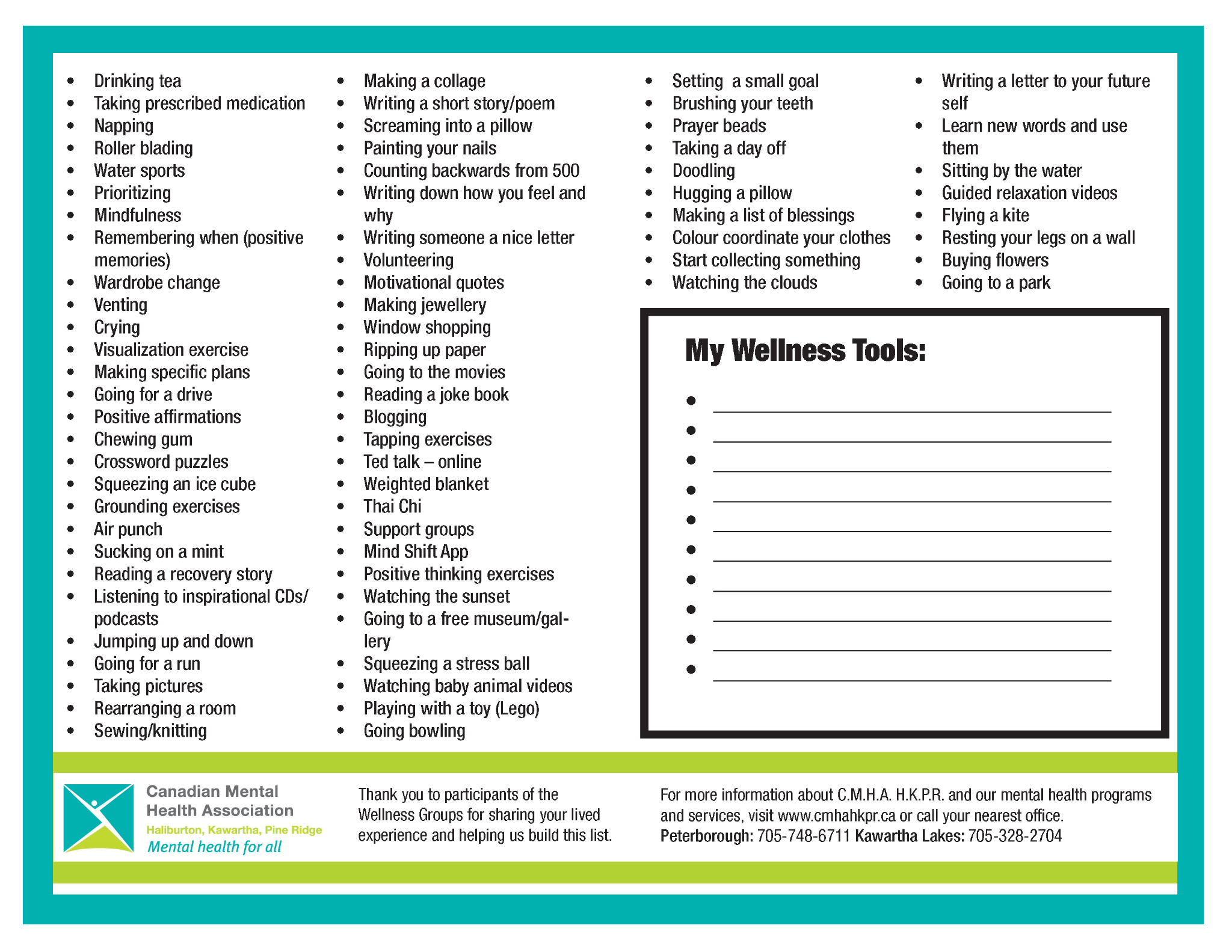

Supplement: Supplementary file 1 [file Table_1.DOCX]
